# How particle shape affects granular segregation in industrial and geophysical flows

**DOI:** 10.1073/pnas.2307061121

**Published:** 2024-01-29

**Authors:** Fernando David Cúñez, Div Patel, Rachel C. Glade

**Affiliations:** ^a^Department of Earth and Environmental Sciences, University of Rochester, Rochester, NY 14627; ^b^Department of Mechanical Engineering, University of Rochester, Rochester, NY 14627

**Keywords:** segregation, Brazil nut effect, armoring, rivers, shape

## Abstract

Industrial and environmental granular flows commonly exhibit a phenomenon known as “granular segregation,” in which grains separate according to physical characteristics (size, shape, density), interfering with industrial applications (cement mixing, medicine, and food production) and fundamentally altering the behavior of geophysical flows (landslides, debris flows, pyroclastic flows, riverbeds). While size-induced segregation has been well studied, the role of grain shape has not. Here we conduct numerical experiments to investigate how grain shape affects granular segregation in dry and wet flows. To isolate the former, we compare dry, bidisperse mixtures of spheres alone with mixtures of spheres and cubes in a rotating drum. Results show that while segregation level generally increases with particle size ratio, the presence of cubes decreases segregation levels compared to cases with only spheres. Further, we find differences in the segregation level depending on which shape makes up each size class, reflecting differences in mobility when smaller grains are cubic or spherical. We find similar dynamics in simulations of a shear-driven coupled fluid-granular flow (e.g., a simulated riverbed), demonstrating that this phenomenon is not unique to rotating drums; however, in contrast to the dry system, we find that the segregation level increases in the presence of cubic grains, and fluid drag effects can qualitatively change segregation trends. Our findings demonstrate competing shape-induced segregation patterns in wet and dry flows that are independent from grain size controls, with implications for many industrial and geophysical processes.

## Significance

Granular materials like cereal, pharmaceuticals, sand, and concrete commonly organize such that grains segregate according to size rather than uniformly mixing. For example, in a jar of nuts, the largest ones are commonly found at the top. Here, we use computer simulations to explore how grain shape controls this phenomenon in industrial and natural settings. We find that even small differences in shape can substantially change the amount and style of segregation, with different effects depending on whether the system is wet or dry. This study demonstrates the importance of grain shape in different systems ranging from food and medicine production to geophysical hazards and processes such as landslides, river erosion, and debris flows on Earth and other celestial bodies.

**Fig. 1.**
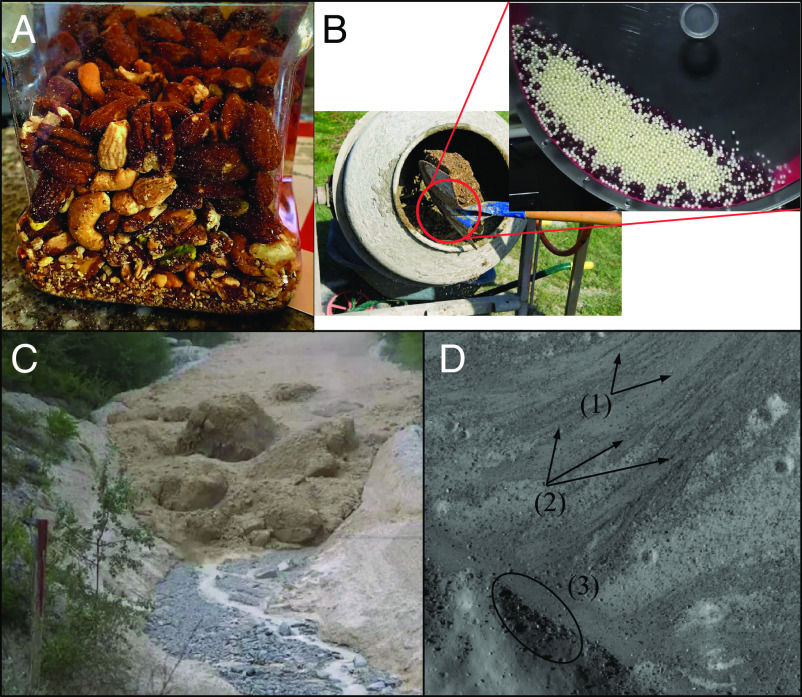
Processes of granular segregation. (*A*) Brazil nut effect in a jar of nuts. (*B*) Process of mixing cement (https://homemasterok.desigusxpro.com/en/pri-kakojj-temperature-mozhno-zalivat-beton.html). *Inset*: Granular mixture in a rotary drum composed by marbles with diameters of $d_{s}=$ 4 and $d_{b}=$ 8 mm. (*C*) Granular segregation in the front of the Illgraben debris flow. Photo by Pierre Zufferey. Image credit: American Geophysical Union. (*D*) Debris flow deposit-terminations in Kepler moon crater (latitude 8.32^°^N, longitude 37.69^°^W) (21). 1) Finer-grained fractions, 2) coarse dark levees, and 3) terminal deposits.

Granular materials are commonly found in our daily lives in a multitude of industrial applications (e.g., cement, pharmaceuticals, food grains) (Fig. 1 *A* and *B*) and in nature (e.g., rocks, sand, snow, soil) (Fig. 1 *C* and *D*). Because they can exist in solid-like and fluid-like states under the influence of external forces, granular materials have no single constitutive equation and we have yet to gain a complete understanding of their complex behavior (1, 2). Further, mixtures of granular materials commonly exhibit an emergent phenomenon known as “segregation” in which grains of different size, shape, density, and roughness self-organize and prevent uniform mixing (3–5). One of the most common examples of granular segregation is the “Brazil nut effect,” which occurs when relatively smaller grains fill in voids beneath relatively larger grains when disturbed, causing larger grains to migrate toward the top of the pile over time (Fig. 1*A*) (6). You have likely experienced this when eating a jar of nuts or pouring cereal into a bowl. Granular segregation can be a severe nuisance, interfering with a variety of mixing processes in the cement, food, and pharmaceutical industries (3).

Granular segregation is also pervasive in nature, where sediment grain size ranges from very fine silt to massive boulders (7). Geophysical flows such as debris flows (Fig. 1*C*) (8), landslides (9), pyroclastic flows (10), and slow-moving, lobate arctic soil patterns (11) exhibit strong segregation, in which large boulders tend to organize at the front of the flow or in levees at the edges, leading to self-channelization that increases runout distance and destructive potential (3, 12, 13). Segregation also occurs for granular beds driven by shear flows, such as wind-blown or subaqueous ripples and dunes (14, 15), beaches (16), and riverbeds where large grains can armor the surface and influence erosion rates and sediment transport dynamics (17, 18). These processes are ubiquitous not only on Earth but on other planetary bodies, including asteroids (19) and planets or moons with a granular surface (Fig. 1*D*) (20–22).

While granular segregation for the simplified case of spherical grains has been extensively studied (23–26), our ability to predict and control its effect in industrial or natural settings is limited; complex interactions between size, density, friction, shape effects, and disturbance rate can lead to unexpected outcomes (3, 27). One of the least explored aspects of segregation is the role of shape, though the presence of nonspherical grains is ubiquitous in most industrial and natural flows (28). Some previous studies have examined the role of grain shape in controlling rotating drum segregation patterns, showing that the presence of angular shapes can dissipate more rotational energy, affecting how grains interact with the wall and with each other (5, 28–30). Grain shape has been shown to alter mobility in a variety of flow regimes, with sharp edges of cubes dissipating energy faster than spheres and decreasing mobility (30–32). However, findings from these studies are often seemingly conflicting and have been difficult to synthesize because it is nontrivial to disentangle the role of shape and size, and different filling levels and rotational speeds used in different studies can result in complex, unpredictable radial segregation patterns that are challenging to compare (4, 31–36). Only recently has a universal framework been proposed for segregation levels with different shapes; Jones et al. (5, 28) found that segregation levels for bidisperse grains (disks, rods, spheres) in a numerical model depends largely on the single-grain volume ratio between the two species rather than diameter ratio, which can be challenging to define for different shapes. According to their results, segregation levels increase logarithmically with volume ratio, and grains with equal volume exhibit zero segregation. This promising work demonstrates that differences in grain volume can account for shape effects on segregation; however, their results show that segregation levels can still vary substantially for different shapes even within the same volume ratio. Many other questions remain, including how angular shapes, shapes that exhibit negative curvature, and the presence of a fluid influence segregation levels.

Here, we use numerical models building in complexity to explore the role of grain shape in controlling granular segregation. First, we examine a partially filled rotating drum filled with dry, bidisperse grains (spheres and cubes) at a low rotational velocity. We choose this setup because it is relevant not only for industrial mixing applications, but also for geophysical flows such as debris flows and landslides (37). We choose to compare spheres with cubes because they are not too dissimilar in shape; thus, our findings may demonstrate how even mild shape differences control segregation, leaving more extreme shapes (long rods, stars, etc.) to future studies. We explicitly control for grain size by comparing results for bidisperse spheres alone with results for bidisperse mixtures of spheres and cubes and find that the presence of cubic grains not only changes segregation levels with respect to the purely spherical case, but introduces new behavior in which segregation levels depend on which shape makes up the small size class. We find that mixtures of small cubes and big spheres experience lower segregation levels than mixtures of big cubes and small spheres at the same size ratio due to shape-induced changes in mobility. Next, we test numerically whether this finding applies in an entirely different system in which fluid shear drives motion over a granular bed (e.g., a riverbed). While we find similar behavior in which the segregation level depends on the shape that makes up the small size class, results show that the presence of fluid drag can qualitatively alter segregation trends, resulting in 1) larger segregation levels in runs with cubic grains for all cases and 2) inverse segregation in which smaller cubes organize at the bed surface. Our work shows that grain shape can exert a fundamental control on segregation, both quantitatively and qualitatively, in simulations of industrial and geophysical flows. These findings demonstrate the need for more attention on grain shape to understand granular dynamics, with implications for efforts to control granular segregation in industry, to predict the behavior of destructive geophysical flows, and to understand sediment dynamics in rivers and windblown dunes that are pervasive on Earth and other planets.

## Grain Shape Controls on Segregation in a Dry Rotating Drum

To isolate the purely granular effects of shape while controlling for size differences, we run dry, bidisperse models in a rotating drum for cases with 1) only spheres or only cubes with varying single-grain volume ratio ($1.3\leq V_{b}/V_{s}\leq 30$), where $V_{b}$ and $V_{s}$ are the volumes of each particle for the big and small species, respectively; 2) mixtures of spheres and cubes varying the single-grain volume ratio ($0.03\leq V_{□}/V_{{}^{\circ}}\leq 30$), where $V_{□}$ and $V_{{}^{\circ}}$ are the volumes of each particle for the cubical and spherical species, respectively. Following refs. 5 and 28, we use volume as a measure of size difference because diameter is not straightforward to define for different shapes. Note that the term “volume ratio” hereafter refers to the volume ratio between single grains of each shape class, not the total volume in the drum. By examining differences in segregation levels and patterns between these cases for the same volume ratios, we can truly isolate the effects of shape.

**Fig. 2.**
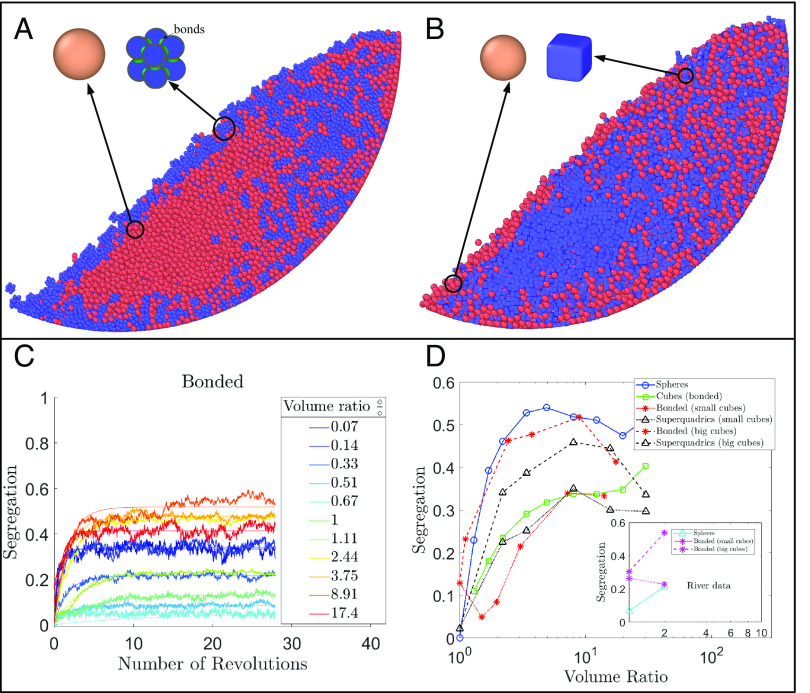
(*A*) Snapshot of particle positions of a mixture of spheres and cubic grains made of bonded spheres ($V_{□}/V_{{}^{\circ}}=3.75$). (*B*) Snapshot of particle positions of a mixture of spheres and cubic grains made of superquadrics ($V_{□}/V_{{}^{\circ}}=0.45$). (*C*) Temporal evolution of the segregation level for mixtures of spheres and cubic grains (bonded spheres) at different single-grain volume ratios. Warming colors indicate increasing volume ratio for cubes versus spheres; values below 1 indicate that cubes are smaller than spheres. Smoothed lines are used to calculate the steady-state segregation level and the time to reach it (Eq. **6**, *Materials and Methods*). (*D*) Steady-state segregation level as a function of the absolute volume ratio for all rotary drum cases. *Inset*: Steady-state segregation level for fluid-sheared granular beds plotted against the absolute volume ratio between grains. In contrast to Fig. 2*C*, the volume ratio here is defined as the volume of a big grain divided by the volume of a small grain, such that all values are greater than or equal to 1. Line colors and symbols indicate different types of grain mixtures; Blue and green denote runs with only one shape (bidisperse spheres and bidisperse cubes, respectively). Black lines indicate runs with bidisperse superquadric grains where cubes are smaller (dotted) or bigger (dashed) than spheres. Red lines indicate runs with bonded particles where cubes are smaller (dotted) or bigger (dashed) than spheres. Note that different segregation levels are seen for the same volume ratio depending on which shape makes up the small size class; for both the dry and fluid cases, runs with small cubes and big spheres exhibit lower segregation levels than the equivalent volume ratio runs with big cubes and small spheres.

We use the open-source code LAMMPS improved for general granular and granular heat transfer simulations (LIGGGHTS), which is based on the discrete element method, to compute granular dynamics. While LIGGGHTS was originally designed to simulate spherical grains, we take advantage of two recently developed capabilities to simulate cubic grains: bonded spheres (Fig. 2*A*) and superquadrics (Fig. 2*B*). Superquadrics allow simulations of near-realistic shapes such as rods, ellipsoids, and more angular shapes such as cubes (albeit with slightly rounded edges). However, state-of-the-art coupled fluid-granular models are not yet able to simulate superquadrics because fluid drag formulations only work for groups of spherical grains (38, 39). Therefore, we also use bonded spheres to create lumpy cubic grains of various sizes, which we hereafter refer to as “bonded cubes,” in order to test whether this approach can be a good approximation for real shapes in fluid simulations. These bonded cubes also allow us to explore the effects of grain shapes with negative curvature (Fig. 2*A*) (40). We calculate the volume of each bonded cube as the total volume of the bonded spheres, plus the volume of the void space in the middle of the grain. We slightly increase the density of each bonded sphere to account for this void space, allowing for equal effective density of bonded cubic grains and other grains (*Materials and Methods*). Cubes and spheres are initially randomly distributed within the drum at equal total volumes between the two species, with a packing fraction of around 30%, and the drum is driven at a low rotational speed representative of a variety of industrial and natural flows. For all cases, we calculate the segregation level once the system has reached a quasi-steady state (Fig. 2*C*) and the time that the mixtures take to reach it (*Materials and Methods*). The segregation level is calculated such that *S* = 0 represents a completely mixed system (equal proportions of both grains), and *S* = 1 represents a completely segregated system (only a single type of grain present) following the method described in ref. 41 (*Materials and Methods*). We choose this segregation metric because it works for mixtures with more than two species, minimizes averaging window size bias[es], and explicitly accounts for different total numbers of grains of each species. We validated segregation calculations by computing the segregation level in a simulation where both species were equal-sized spheres, finding that the segregation level through time was zero (*SI Appendix*). We also validated the model setup by comparing results with a physical experiment in a rotating drum, using the same rotation rate and marbles of the same size (Fig. 1*B* and Movie S1).

Our results illuminate the importance of both grain size and shape in controlling segregation, clearly demonstrating that shape alone can substantially affect segregation levels. We observe similar qualitative behavior for all runs; Fig. 2*C* shows the evolution of the segregation level for mixtures of spheres and bonded particles, where cooler colors correspond to small volume ratios and warmer colors to large volume ratios. In all cases, the segregation level starts at zero, where the particles are randomly distributed and then increases until it reaches a steady state (see *SI Appendix* for other time series). In agreement with previous studies (5, 28, 42), the steady-state segregation level for all cases tends to increase with volume ratio for ratios up to about 10 (Fig. 2 *C* and *D*). Big grains, regardless of shape, tend to migrate toward the surface and walls of the drum. However, once volume ratios reached about 10 or higher, we could no longer define well-segregated regions in the mixture. This coincides with the onset of a segregation inversion in which big grains begin to accumulate at the center of the drum (*SI Appendix*). This result agrees with previous studies that found inverse segregation for large size ratios, where depending on the roughness of the walls and the weight of the grains, big grains may concentrate in the center of the drum (32, 43, 44).

The effect of grain shape can be seen in substantial quantitative differences in segregation levels in all runs even at the same volume ratios. The presence of cubic grains decreases segregation level in all studied cases, except for the case of equal volume ratio (volume ratio = 0) in which nonspherical grains produce segregation levels about 10% higher, in contrast to previous findings (5, 28) (Fig. 2*D*). Runs with superquadric cubes alone exhibit nearly half the segregation levels as spheres alone. However, the most interesting result is found for mixtures of cubes and spheres. We observe shape-induced differences in segregation trends, where segregation levels are lowest for cases in which cubes are smaller than spheres, and higher for cases in which cubes are larger than spheres for the same volume ratio; this occurs for both superquadric and bonded cubes (Fig. 2*D*). While runs with big bonded cubes and small spheres are nearly identical to runs with spheres alone, runs with bigger superquadric cubes experience less segregation. The lowest segregation levels occur for cases with small superquadric or bonded cubes mixed with bigger spheres.

Our results illustrate a clear, significant grain shape control on segregation in granular flows independent from the role of grain size. But why do we observe substantial differences in the segregation level with different shapes? In typical rotating drum configurations, most of the segregation occurs in the avalanching flow layer at the surface, whereas the center of the drum rotates as a solid-like body and experiences little to no segregation (26, 45). Therefore, one would expect a decrease in segregation to correspond to lower grain mobility (46) due to a lower shear rate or a deeper or more densely packed avalanche layer (26, 45). To unravel shape-induced differences in segregation, we analyze average particle velocities, packing fraction, and avalanche depth for each case in both the flowing surface layer and the solid-like inner layer (*SI Appendix*). Our findings show no clear trends that would explain the observed differences in segregation levels. For example, runs with the highest segregation level—spheres alone, and bonded big cubes with small spheres—exhibit the lowest and highest packing fraction, respectively, while runs with the lowest segregation level have intermediate packing fractions (*SI Appendix*, Fig. S9). The depth of the flowing layer similarly shows no clear relationship to the segregation level (*SI Appendix*, Fig. S7). Similarly, the highest segregation runs tend to exhibit lower average velocities than the low segregation runs, in both the flowing and solid-like layers (*SI Appendix*, Fig. S10). These puzzling findings point toward a distinct shape-controlled mechanism for the observed differences in segregation level. While future work is needed to fully understand this mechanism, here we discuss a possible explanation.

**Fig. 3.**
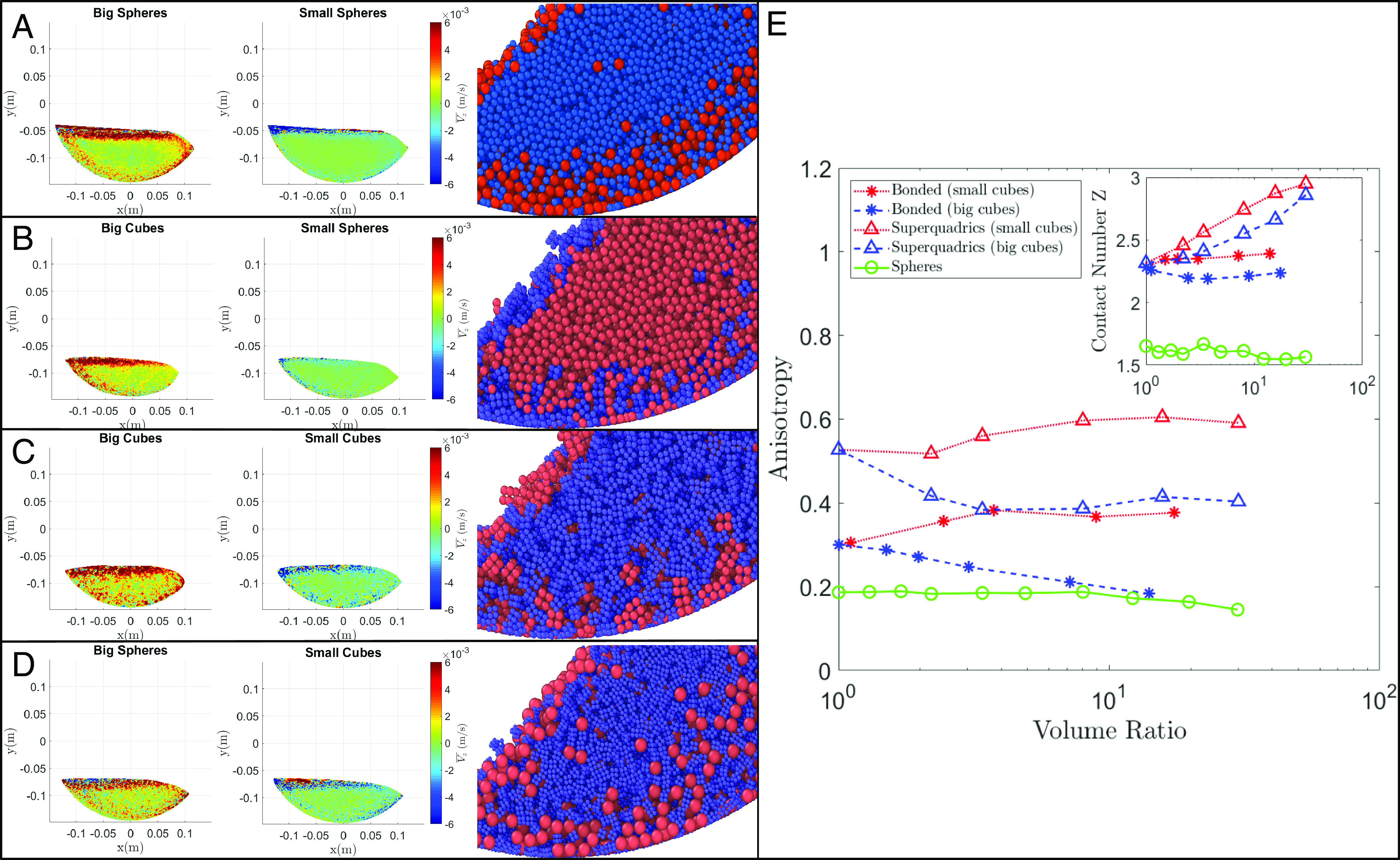
(*A*–*D*) Time-averaged surface-normal (“vertical”) velocities for big and small species and instantaneous snapshots of particle positions of a mixture of (*A*) spheres of different sizes with a volume ratio of $V_{b}/V_{s}=3$; (*B*) big cubes and small spheres with a volume ratio of $V_{□}/V_{{}^{\circ}}=3$; (*C*) cubes of different sizes with a volume ratio of $V_{\mathrm{big}}/V_{\mathrm{small}}=3$; and (*D*) big spheres and small cubes with a volume ratio of $V_{{}^{\circ}}/V_{□}=3$. (*E*) Anisotropy as a function of the ratio of volume. *Inset*: Mean contact number per particle Z as a function of the volume ratio.

Turning to videos of each model run, we observe qualitatively different dynamics in each case depending on the different shapes present. In cases with the highest segregation levels (i.e., where the small grains are spheres), big grains are efficiently carried up the drum wall to be re-exposed at the surface, maintaining a clear separation in which big grains inhabit the outer layer while small grains lie on the inside (Fig. 3 *A* and *B*, images on *Right*). In contrast, in cases with a lower segregation level (i.e., where the small grains are cubes), many of the big grains experience upward trajectories toward the center of the drum before they can be carried back up to the surface (Fig. 3 *C* and *D*, images on *Right*), leading to a more mixed steady state with lower segregation levels. Snapshots of time-averaged surface-normal (“vertical”) velocities across the drum further support this idea, illustrating that big grains experience higher upward-directed vertical velocities and small grains experience higher downward-directed vertical velocities in the center of the drum for low segregation level cases than for high segregation level cases (Fig. 3*A*–*D*). In essence, the presence of small cubes activates the center of the drum, leading to more rearrangement of grains and therefore a lower segregation level at steady state. While time-averaged data are noisy, plots of mean vertical velocity show that large particles tend to rise faster for cases with small cubes; even more clearly, small cubes tend to sink faster than small spheres in most runs (*SI Appendix*, Fig. S8).

Qualitatively, we observe in the videos that small cubes organize into aligned chains of grains near the wall that minimize shear from the wall; as the drum rotates, they sporadically rearrange and effectively act as pole vaults, lofting the big grains upward into the center of the drum, whereas small spheres constantly shift and rearrange in a more continuous fashion that keeps the big grains closer to the wall (Movies S5–S8). This may point toward shape-driven changes in the efficiency of squeeze expulsion (47). The qualitative observation of higher self-organization with small cubes is supported by measurements of fabric anisotropy and contact number for each case (Fig. 3*E*). Fabric anisotropy occurs when shear strength and dilatancy exhibit different values along different directions due to the state of the granular material’s microstructure, where microstructure refers to the arrangement of particles, void spaces, and interparticle contacts (48). Here, we characterize microstructure with a fabric tensor based on inter-particle contacts due to forces being transmitted along these contacts, forming force chains (see *Materials and Methods* for the mathematical formulation of the fabric tensor). All cases with cubes exhibit higher anisotropy than for spheres alone; cases with small cubes and big spheres have higher anisotropy than cases with big cubes and small spheres; and superquadric cubes exhibit higher anisotropy than bonded cubes. This result is possibly due to the negative curvature of bonded cubes, such that they can fit together in a variety of ways, whereas superquadrics preferentially align face to face. The mean contact number for all cubic runs is also substantially higher than that of spherical runs (Fig. 3*E*, *Inset*); cases with small cubes experience a higher contact number than for big cubes, and superquadrics experience higher contact numbers than bonded cubes. We interpret these results to show that the presence of small cubes effectively decreases the shear rate in the drum, decreasing segregation efficiency and allowing more mixing in the center of the drum.

Our results demonstrate the importance of the small size fraction in controlling segregation. This aligns with a commonly observed shear-induced percolation mechanism in the presence of gravity (47); thus, we anticipate that the shape of the smallest size fraction may be the dominant factor for shape-controlled segregation. Analysis points toward a possible mechanism for shape-controlled segregation, in which different styles of grain rearrangement in the center of the drum lead to differences in segregation level. Further work is needed to quantitatively diagnose this behavior.

Our results also show that superquadric and bonded cubes exhibit similar segregation behaviors. In the next section, we use bonded cubes in a coupled fluid-granular simulation to show that shape-controlled segregation also occurs in an entirely different system: shear flow over a granular bed (e.g., a riverbed).

## Grain Shape Controls on a Fluid-Sheared Granular Bed

Fluid shear flow over granular beds sculpts planetary landscapes, as wind creates ripples and dunes and rivers transport sediment, carving mountain ranges and delivering nutrients to the ocean. Granular segregation is ubiquitous in these types of flows, especially in rivers or on beaches where big grains commonly armor the bed surface. This armoring can change the morphology and dynamics of the flow, with implications for flooding, erosion, and landscape evolution processes (16, 17). It is thought to occur due to a variety of processes, including preferential removal of fine grains due to sediment supply limitations (49) and granular segregation via the Brazil nut effect as grains are disturbed by fluid near the surface of the bed (18, 50) and experience creep at slower rates deeper into the bed (51). However, most formulations of bedload transport in rivers assume spherical grains that do not represent natural sediment. Only recently has grain shape been shown to affect fluvial sediment transport via changes in fluid drag (52, 53) and interactions with the granular bed (52, 54). The role of grain shape in controlling granular segregation processes in natural fluid flows has been unexplored.

**Fig. 4.**
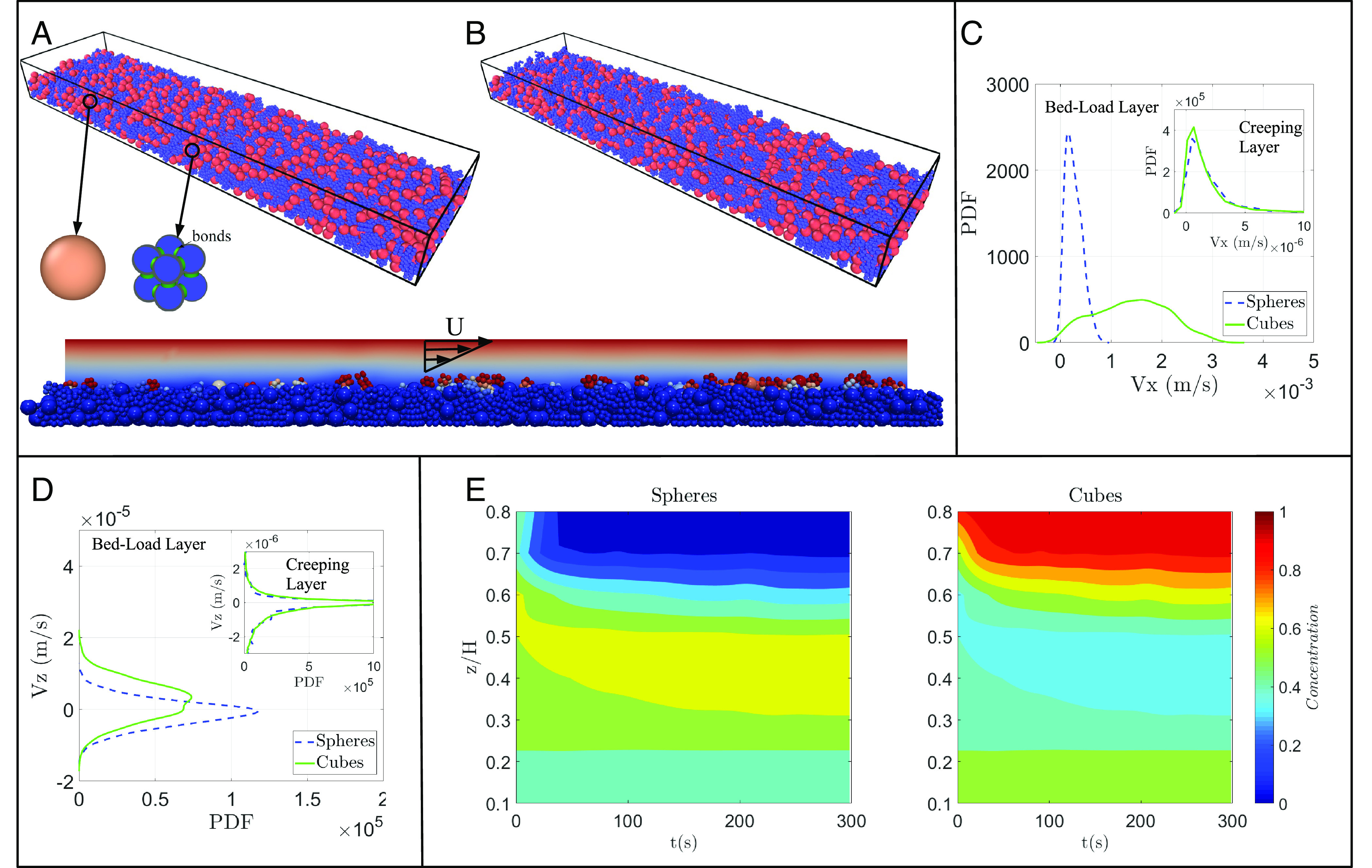
Numerical river data. Snapshots of particle positions of a mixture of spheres and cubic grains developed by bonded spheres ($V_{□}/V_{{}^{\circ}}=0.5$) at (*A*) initial condition (t = 0 s) and (*B*) steady state (t = 300 s). (*C*) PDF of downstream velocities in the flowing and creeping (*Inset*) layers for each species of particles. (*D*) PDF of vertical velocities in the flowing and creeping (*Inset*) layers for each species of particles. (*E*) Temporal evolution of concentration of spheres and cubic grains as a function of the channel height.

To begin to explore grain shape effects on segregation in natural systems, we run simulations of a Couette flow in a rectangular channel with bidisperse spheres and bonded cubes (Fig. 4*A*), tracking segregation of the bed and grain velocities through time. We use the coupled fluid dynamics/discrete element method (CFDEM) modeling software, which couples the LIGGGHTS granular dynamics and OpenFOAM fluid dynamics models (55), to observe laminar flow over a granular bed in a rectangular channel with periodic boundary conditions in the streamwise direction. The flow velocity is set to be just above the threshold of motion ($\theta /\theta_{\mathrm{cr}}\approx 1.5$) for the biggest grains in the channel (*Materials and Methods*). We choose to use laminar flow for simplicity, in order to focus on first-order interactions between fluid and grains; while future studies may examine the role of turbulence characteristic in many natural flows, studies have shown that sediment transport in laminar flows is fundamentally similar to that of turbulent flows (56).

Our results show that granular segregation driven by fluid shear exhibits similar behavior to that seen in the dry rotating drum in which segregation level depends on which shape makes up the small size class (Fig. 2*D*, *Inset*), suggesting that our findings are not unique to that system. Runs with small cubes and big spheres experience only a third of the segregation level seen for runs with big cubes and small spheres. However, we find that the effects of fluid–grain interactions can lead to both quantitative and qualitative differences in segregation trends. In contrast to the rotating drum case, the presence of cubes leads to higher segregation levels than spheres alone (Fig. 2*D*, *Inset*); further, cubes always organize at the top of the bed, even when they are smaller than the spheres. This can be seen for the case shown in Fig. 4, in which $V_{□}/V_{{}^{\circ}}=0.5$. Beginning from a fully mixed state (Fig. 4*A*), the smaller blue cubes preferentially organize at the bed surface through time (Fig. 4 *B* and *E*). This demonstrates a shape-induced inverse segregation in natural flows that may offset the armoring phenomenon.

Why do we observe qualitatively different segregation trends in the presence of a fluid? Probability density functions (PDFs) of grain velocities show that cubes experience faster instantaneous downstream velocities than spheres (albeit with much larger variability)(Fig. 4*C*) and upward directed vertical velocities (Fig. 4*D*), while spheres subtly tend toward downward directed vertical velocities. We can better understand this behavior by examining the concentration of each grain shape with respect to the total number of grains in a series of layers at different depths in the bed at the end of the model run (Fig. 4*E*). At t = 0, grains are randomly mixed throughout the bed. As time progresses, they experience rapid segregation in which cubes accumulate at the bed surface (approx. where z/H = 0.7). A zone of low cube concentration grows through time with depth just beneath the surface; in contrast, spheres accumulate just beneath the bed surface in a concentrated layer that grows in depth over time.

We interpret these results to illustrate the role of the fluid in driving segregation patterns in a granular bed. Because cubes experience a higher drag force than spheres (52, 57), once they reach the surface, they can move faster and are more likely to continue moving. This likely prevents them from settling back into the bed, decreasing their ability to percolate downward and causing them to collect on the bed surface even if they are smaller than the spheres, leading to higher segregation levels. At depth, however, grain–grain interactions dominate, causing spheres to migrate upward and collect just beneath the surface above which fluid effects take over (see high concentrations of spheres at z/H = 0.4 to 0.5). Note that any purely granular controls in the subsurface are small, as illustrated in the similar PDFs of vertical velocity in Fig. 4*D*, *Inset*, highlighting the importance of the fluid which dominates over grain–grain interactions.

These findings point toward the need for further exploration of the role of fluid effects in nonspherical granular flows and may begin to explain enigmatic observations in riverbeds, where in some situations big grains armor the surface, while in other situations, finer grains are found at the top (58, 59). Further work is needed to determine whether our findings apply to natural rivers, where dense sediment of many different shapes is found in turbulent flows.

## Discussion

Our findings show that grain shape cannot be ignored in granular segregation processes, even when size effects are accounted for. Shape-induced segregation trends can vary both quantitatively and qualitatively depending on competition between grain–grain and grain–fluid effects. In dry flows, we observe behavior in which small cubic grains can experience high anisotropy and contact numbers, counterintuitively leading to more mixing in the center of the drum and therefore a lower segregation level. This finding has possible implications for industrial applications where segregation is a nuisance. While we see similar shape-dependent segregation behavior in fluid shear-driven slows, cubic grains of any size instead increase segregation levels compared to spheres alone; fluid–grain interactions can even lead to qualitative shifts in behavior, producing an inverse percolation-driven segregation in which small cubes accumulate at the surface. The presence of fluid also magnifies the importance of segregation in the flowing layer, while in the dry case, grain motions near the drum wall also contribute to differences in segregation level. These results illuminate competing segregation effects due to grain–grain and grain–fluid interactions, which could lead to different qualitative behavior depending on the total volume fraction and inertial regime of different industrial and geophysical flows.

Our methods demonstrate a way to isolate the role of grain shape from size disparities by comparing results for the same volume ratio with different shape combinations. Future studies can use this approach to examine different shapes, mixtures with more than two grain classes, and to see whether our results hold for rotating drums with different rotation rates and filling levels. Studies can also explore whether our results can be harnessed in industrial applications to decrease segregation levels in mixing processes by adding nonspherical grains to mixtures. While our analysis suggests that small grains are inherently important to segregation processes, further studies could explore whether it is the size or abundance of cubic grains that most strongly controls segregation; because we use an equal total volume of each species in our models, small grains are more abundant than big ones. The fact that runs with big superquadric cubes exhibit lower segregation levels than those with spheres alone illustrates that even small numbers of cubes can have an effect on segregation dynamics. It is possible that experiments with abundant big cubic grains could experience effects similar to those we see for small cubes.

Grain shape–induced differences in segregation imply shape controls on bulk rheology as well (46, 60), with implications not only for industry but also for geophysical flows. A recent study demonstrated that debris flow rheology is controlled by the solid volume fraction and therefore the distance to the jamming transition (61). Since debris flows are also thought to be strongly controlled by granular segregation (62), accounting for shape in debris flow modeling could be doubly important. Another recent study found that the temporal evolution of angular grains in a pyroclastic flow determines flow rheology (63). Indeed, changes in packing fraction known to affect rheology have also been shown to result in qualitative shifts in segregation trends (64). In light of these studies and our findings, we suggest that grain shape exerts a fundamental control on both the segregation and rheology—and therefore destructive potential—of geophysical flows. While our fluid shear-driven model applies to riverbeds, beaches, and possibly windblown settings—examples of dilute suspensions where the volume of moving sediment is low compared to the volume of the fluid (2)—future work could explore whether similar competition between shape-induced grain–grain and grain–fluid controls on segregation applies in industrial and natural systems that behave as dense suspensions (65), such as cement mixers (66), debris flows and landslides (2). Further work could explore shape-induced granular segregation processes in noninertial systems over longer timescales, such as hillslopes that evolve through slow soil creep or crystal segregation in magmas (67).

## Materials and Methods

### Model Description.

In our numerical simulations for the purely granular effects, we used the open-source code LIGGGHTS (68, 69) and its modified version that includes bond equations (38) to compute the interactions of each individual particle and the wall by solving the linear and angular momentum equations, given by Eqs. **1** and **2**, respectively:

[1] $$\begin{array}{cc} m\frac{d\vec{u}}{\mathrm{dt}} & =\sum_{i\neq j}^{N_{c}}{\vec{F}}_{c,ij}+\sum_{i}^{N_{w}}{\vec{F}}_{c,iw}+m\vec{g},\\ I\frac{d\vec{\omega }}{\mathrm{dt}} & =\sum_{i\neq j}^{N_{c}}{\vec{T}}_{c,ij}+\sum_{i}^{N_{w}}{\vec{T}}_{c,iw}, \end{array}$$

where $\vec{g}$ is the acceleration of gravity and, for each solid particle, m is the mass, $\vec{u}$ the velocity, I the moment of inertia, $\vec{\omega }$ the angular velocity, $\vec{F_{c}}$ the resultant of contact forces, and $\vec{T}$ the resultant of contact torques. The indices in $F_{c}$ and T correspond to the collisions between particles i and j, and between particle i and the wall w.

To compute the contact forces between particles ${\vec{F}}_{c,ij}$ and between particles and the rotational wall ${\vec{F}}_{c,iw}$, we use the Hertzian contact theory (70) which consists of a system with two springs to represent the normal and tangential forces acting between two spheres colliding. The DEM parameters used in this work are taken from previous studies (18, 39) and are detailed in Table 1.

**Table 1.** DEM simulation parameters

Particle density ρ (kg/m^3^)	1,190
Young’s modulus E (MPa)	5
Poisson ratio σ	0.45
Particle–particle friction coefficient $\mu_{p}$	0.5
Particle–wall friction coefficient $\mu_{w}$	0.5
Coefficient of restitution ϵ	0.5
Time step ΔT (s)	1×10^−6^
Angular velocity of the drum Ω (rpm)	12

For the fluid-sheared granular bed, the computations were carried out by using the open-source code CFDEM (55), that couples LIGGGHTS (described previously) and OpenFOAM (which computes the fluid motion in an Eulerian frame). For this case, the LIGGGHTS code solves a modified Eq. **1**, where we add the fluid contributions given by ${\vec{F}}_{D}+{\vec{F}}_{\mathrm{stress}}+{\vec{F}}_{\mathrm{am}}$ in the right-hand side, where ${\vec{F}}_{D}$ is the drag force caused by the fluid on particles, ${\vec{F}}_{\mathrm{stress}}=V_{p}\left[-\nabla P+\nabla \cdot \overset{¯}{\overset{¯}{\tau }}\right]$ is the force caused by the fluid stresses, and ${\vec{F}}_{\mathrm{am}}$ is the added mass force which is important for simulations involving liquids (39). P is the fluid pressure and $\overset{¯}{\overset{¯}{\tau }}$ is the deviatoric stress tensor of the fluid. On the other hand, OpenFoam computes the conservation of mass and momentum of the fluid by the following equations:

[2] $$\begin{array}{c} \frac{\partial \rho_{f}\varepsilon_{f}}{\mathrm{dt}}+\nabla \cdot \left(\rho_{f}\varepsilon_{f}{\vec{u}}_{f}\right)=0,\;\;\;\;\;\;\\ \frac{\partial \rho_{f}\varepsilon_{f}{\vec{u}}_{f}}{\mathrm{dt}}+\nabla \cdot \left(\rho_{f}\varepsilon_{f}{\vec{u}}_{f}{\vec{u}}_{f}\right)=-\varepsilon_{f}\nabla P+\varepsilon_{f}\nabla \cdot \overset{¯}{\overset{¯}{\tau }}-\frac{{\vec{F}}_{D}}{V_{\mathrm{cell}}}, \end{array}$$

where ${\vec{u}}_{f}$ is the velocity of the fluid phase, ε is the volume fraction of the fluid in a calculation cell, and $V_{\mathrm{cell}}$ is the volume of the considered calculation cell. The estimations of the drag force ${\vec{F}}_{D}$ imposed on each particle come from experimental correlations based in the flow regime and the volume fraction (71). The CFD parameters used in this work are detailed in Table 2.

**Table 2.** CFD simulation parameters

Fluid density $\rho_{f}$ (kg/m^3^)	1,050
Fluid viscosity $\mu_{f}$ (mPa s)	72.2
Top mean velocity U (m/s)	0.02
Mean fluid height $h_{f}$ (m)	0.004
Time step $\Delta T_{f}$ (s)	5×10^−5^
Channel dimensions X,Y,Z (m)	0.1×0.025×0.01

With the conditions described above, the Reynolds number $Re_{f}=$$\rho_{f}Uh_{f}/\mu_{f}$ is around 1.5 that assures the flow is in a laminar regime. The Shields number $\theta =\frac{\mu_{f}U/h_{f}}{\left(\rho_{p}-\rho_{f}\right)gd_{p}}$ has values ranging from 0.13 to 0.18 (depending on the size of particles), and the threshold of motion for this case is $\theta_{\mathrm{cr}}\approx$ 0.1 (51, 72).

### Numerical Setup and Validation.

For the particles, we used: i) spheres with sizes varying from 1.5 to 4.5 mm; ii) cubical particles formed from bonded spheres, that were implemented numerically by placing into permanent contact eight spheres, that do not overlap with each other, with bonds half the diameter of spheres and being considered solid, as shown in Fig. 2*A*. In order to prevent any gravitational stratification, we match the mass of the eight bonded spheres to the solid spheres to estimate the density of individual grains that composed a bonded cube; and iii) cubical particles formed from superquadric shapes (Fig. 2*B*) which are determined by the following equation:

[3] $$\begin{array}{c} {\left({\left|\frac{x}{a}\right|}^{n_{2}}+{\left|\frac{y}{b}\right|}^{n_{2}}\right)}^{\frac{n_{1}}{n_{2}}}+{\left|\frac{z}{c}\right|}^{n_{1}}-1=0, \end{array}$$

where a, b, and c are the lengths of the particles’ semi-axis, and $n_{1}$ and $n_{2}$ determine the particle shape and the surface blockiness (5, 28, 73). To obtain the cubical particle shown in Fig. 2*B*, we set $n_{1}$ and $n_{2}$ equal to 8.

For the case of the purely granular interactions, we consider a rotary drum with a diameter D of 0.3 m and a width W of 0.05 m driven by a rotational speed of 12 RPM for 140 s; meanwhile, for the case of the fluid-sheared granular bed, we used a rectangular channel with dimensions of 0.1 m in the streamwise direction, 0.025 m in the cross-stream direction, and 0.01 m in depth; where we imposed a velocity at the top wall of 0.02 m/s for 300 s. For both cases, two species of particles were randomly placed in equal total volume ratios. In order to run the numerical simulations, first we let the mixture of particles to settle for 1 s and to rest for another 1 s.

As part of the validation of our dry model, we also carried out a physical experiment with a rotary drum filled with glass beads of various sizes [see *SI Appendix* (74) for a video comparing the experiments and numerical simulations].

### Calculation of Segregation Levels.

Fig. 2*C* shows the evolution of segregation level for mixtures of spheres and bonded particles, where cooler colors correspond to small volume ratios and warmer colors to large volume ratios. In all cases, the segregation level starts at zero, where the particles are randomly distributed and then increases until it reaches a steady state. For each case, we fitted the curves of the temporal evolution of the segregation level by using the following expression:

[4] $$\begin{array}{c} S\left(t\right)=S_{f}\left(1-e^{-t/t_{s}}\right), \end{array}$$

where $S_{f}$ is the segregation level at the steady state, t is time, and $t_{s}$ is the time that a case takes to reach the steady state from its initial condition. By fitting the curves shown in Fig. 2*C*, the steady-state level and the time of segregation for each case were determined.

The segregation level that a system reaches is an important parameter to estimate the steady-state behavior of a mixture of particles; however, it is an empirical parameter that varies with the local domain, number of species, and the distribution of particle size. Although there are several studies that focus on determining the segregation level, calculations are inherently biased depending on the choice of window size. To quantify the segregation level, we calculated the fraction of each species with respect to the total number of particles throughout the entire domain, based on dividing the rotary drum in sub-domains as shown in ref. 41. This formulation is useful because it can be applied to systems with any number of different species, rather than being limited to bidisperse systems. Based on an exhaustive analysis of the number of subdomains needed in the rotary drum, we found that the size of a subdomain is best determined by the sum of the sizes of each species [see *SI Appendix* for the study of subdomain sizes].

The domain of the drum is divided into M number of subdomains of rectangular shape to estimate the segregation level of Q types of species present in the mixture. For our study, we consider a distribution of equal total volume ratio for the granular bed, such that the domain does not contain the same number of particles of each species. Therefore, we use a correction factor to determine the fraction of one species with respect to the highest number of particles relative to the other species is given by the following equation (41):

[5] $$\begin{array}{c} P_{\mathrm{ki}}=\frac{n_{\mathrm{ki}}f_{k}}{\max\left(\left(n_{1i}f_{1}\right),\left(n_{2i}f_{2}\right),...,\left(n_{\mathrm{Qi}}f_{Q}\right)\right)}\leq 1, \end{array}$$

where $n_{\mathrm{ki}}$ is the number of particles of the kth species in the subdomain i, and $f_{k}$ is the factor of participation based in the total number of particles of each species given by

[6] $$\begin{array}{c} f_{k}=\frac{\max\left(\sum_{i=1}^{M}n_{1i},\sum_{i=1}^{M}n_{2i},...,\sum_{i=1}^{M}n_{\mathrm{Qi}}\right)}{\sum_{i=1}^{M}n_{\mathrm{ki}}}. \end{array}$$

The instantaneous segregation level S is obtained from the arithmetic mean of the individual fractions of each species of particles k in all M subdomains and is calculated by the following equation:

[7] $$\begin{array}{c} S=1-\left(\frac{1}{N}\sum_{i=1}^{M}\left[\frac{1}{Q-1}\left(\sum_{k=1}^{Q}P_{\mathrm{ki}}-1\right)\sum_{k=1}^{Q}\left(n_{\mathrm{ki}}\right)\right]\right), \end{array}$$

where N is the total number of particles in the mixture. Eqs. **7**–**9** essentially quantify the mean segregation level of the drum while correcting for different total numbers of grains of each type. The resulting segregation level gives a value of 0 for a fully mixed system and a value of 1 for a fully segregated system.

### Calculation of Anisotropy.

The amount of anisotropy that a granular system exhibits is determined by the contact fabric tensor $\hat{R}$, which is calculated by the following equation:

[8] $$\begin{array}{c} \hat{R}=\frac{1}{N_{c}}\sum_{i\neq j}\frac{\vec{r_{\mathrm{ij}}}}{|\vec{r_{\mathrm{ij}}}|}⊗\frac{\vec{r_{\mathrm{ij}}}}{|\vec{r_{\mathrm{ij}}}|}, \end{array}$$

where $\vec{r_{\mathrm{ij}}}$ is the contact vector from the center of particle i to the interparticle contact between particles i and j, ⊗ is the vector outer product, and $N_{c}$ is the total number of particles with at least two contacts. The dimensionless fabric anisotropy tensor $\hat{\mathrm{AF}}$ is proportional to the deviatoric part of the contact fabric tensor $\hat{R}$ and can be estimated by the following expression (74):

[9] $$\begin{array}{c} \hat{\mathrm{AF}}=\frac{5}{2}\left(3\hat{R}-\hat{I}\right), \end{array}$$

where $\hat{I}$ is the identity tensor. Finally, the amount of anisotropy that a system shows AF is given by the norm of the dimensionless fabric anisotropy tensor and can be computed by

[10] $$\begin{array}{c} AF=\sqrt{\hat{\mathrm{AF}}:\hat{\mathrm{AF}}}. \end{array}$$

## Supplementary Material

Appendix 01 (PDF)

Movie S1. Validation of the numerical model with an experiment in real time

Movie S2. Numerical model with a mixture of spheres and bonded spheres for the case *V*_□_/*V*_ᴏ_ = 0.07

Movie S3. Numerical model with a mixture of spheres and cubical superquadrics for the case *V*_□_/*V*_ᴏ_ = 0.06

Movie S4. CFD-DEM model of a granular bed composed by spheres and bonded particles and driven by a Couette laminar flow.

Movie S5. Zoom in of a mixture of spheres of different sizes with a volume ratio of 12.

Movie S6. Zoom in of a mixture of cubes of different sizes with a volume ratio of 12.

Movie S7. Zoom in of a mixture of spheres and cubes of different sizes with a volume ratio of *V*_□_/*V*_ᴏ_ = 12.

Movie S8. Zoom in of a mixture of spheres and cubes of different sizes with a volume ratio of *V*_□_/*V*_ᴏ_ = 0.08.

## Data Availability

All data and code used in this study are available at Fernando David Cúñez, Div Patel, Rachel C. Glade (2023), Dataset for “How particle shape affects granular segregation in industrial and geophysical flows,” Mendeley Data, V1, 10.17632/xchtmc2pp8.1 (75).
