# Supplementary material for: How particle shape affects granular segregation in industrial and geophysical flows
Source: Proc Natl Acad Sci U S A. 2024 Jan 29;121(6):e2307061121. doi: 10.1073/pnas.2307061121 (PMC10861863; doi:10.1073/pnas.2307061121)
Supplement: Supplementary file 1 — Appendix 01 (PDF) [file pnas.2307061121.sapp.pdf]

Supporting Information for

How particle shape affects granular segregation in industrial and geophysical flows

Fernando David Cúñez, Div Patel, and Rachel C. Glade

Corresponding Author name.
E-mail: rachel.glade@rochester.edu

This PDF file includes:

Supporting text
Figs. S1 to S11
Tables S1 to S3
Legends for Movies S1 to S8

Other supporting materials for this manuscript include the following:

Movies S1 to S8

Supporting Information Text

In this document we provide additional information for key aspects of the manuscript. In section I, we show the validation of our numerical model with an experimental setup with similar dimensions. In section II, we provide some information about how we determine the window size to compute the segregation level. In section III, we show instantaneous snapshots of the particles' final state for all the cases we used to produce our dry rotating drum results. In section IV, we show instantaneous snapshots of the final state of force networks for all the cases we used to reproduce the results described in section III.

I. Validation of the numerical model. We carried out an experiment to qualitatively validate our numerical model. The experimental setup (Fig. S1i) consisted of an acrylic drum with a diameter of $D = 30$cm, a stepper motor driver which rotates the drum and is controlled by an Arduino UNO through the GRBL project (https://github.com/grbl/grbl), a set of LED lamps, and a DSLR Nikon D7500 camera. The camera and the motor driver were automatically synchronized to record and save the movies. For this specific case, we placed small marbles ($d_s =$4mm, yellow color in Fig. S1ii) at the bottom and then the bigger ones ($d_b =$8mm, red color in Fig. S1ii) just above the smaller ones. We used this initial condition in order to simulate the exact initial condition for the experiment and the numerical model; however, note that it differs from the initial condition in our main model results, which begin with a fully mixed system. This initial condition would be very difficult to implement in a physical experiment. Finally, we ran the experiment for 140s at a rotation speed of 12RPM (similar conditions to the simulations carried out for the manuscript). From Figure S1ii and movie S1, we found that the behavior of the experiment and the numerical model is highly similar in terms of segregation levels and time to reach a final segregated state.

II. Determination of the window size for segregation level calculations. We studied the influence of the size of the subdomains detailed in Figure S2 in the calculation of the segregation level of the system. The segregation level is strongly dependent on the number and the size of subdomains we have in the system. Even though many studies focused in different techniques to study either level of segregation or mixing, there is no a defined recipe to follow when picking the correct window size. For our study we divided the domain into small squared sections in the x and y directions ($\Delta = dx = dy$) throughout the width of the drum, then we varied the size of Δ from $0.25(d_s + d_b)$ (where the window is around half of the size of one species) to $10(d_s + d_b)$ (where the window is around 20 times the size of one species).

Figure S3 shows the trends of the temporal evolution of the segregation level for different window sizes. In the case of a very small size ($\Delta =0.25(d_s + d_b)$), the segregation level is very high because we are looking at a region where only one particle can fit inside, having thus a totally segregated system in that subdomain ($S \approx 1$). For the case of a big size ($\Delta =10(d_s + d_b)$), the region is big enough that we can encounter both species in same proportions, having thus a totally mixed system in that subdomain ($S \approx 0$). However, for the cases where size is close to the sum of both species diameters ($\Delta \approx 1(d_s + d_b)$), the behavior of the evolution of the segregation level is similar.

To identify which size is better to compute the segregation level we have two references, 1) the initial condition for all the cases is that particles are randomly distributed which S should be around zero, and 2) the segregation level for spheres of equal size ($d_s = d_b = 1$) is zero as the system does not have either segregation by size or shape. Therefore, the best window size to compute the level of segregation is $\Delta = 1(d_s + d_b)$, because all the cases start in the same point at t=0s (Fig S3(d)) and the results are consistent with what we see qualitatively from the particle positions snapshots (Fig S5). Finally, to get the data that are in Figure 2 of the manuscript, we shifted the curves to the zero reference that is given by the case of V_b/V_s=1.

Figure S4 shows the temporal evolution of the segregation level for all the tested cases and their exponential fits.

III. Final state of particles position for all the tested cases. Figure S5 shows instantaneous snapshots of particle positions for size segregation in both cases spheres and cubes.

Figure S6 shows instantaneous snapshots of particle positions for size and shape segregation in for cubes made by bonded particles and superquadrics.

IV. Determination of the depth of the flowing and solid-like layers in the rotating drum. To determine either the flowing layer or the solid-like layer in the rotating drum, first, we rotated the Cartesian coordinates of the particle positions in order to obtain the avalanche surface normal to the vertical axis. We calculated the time average velocity of the assembly of particles for all the cases studied in this work (color map in Figure S7(a)). From that, we horizontally averaged the color-map in order to obtain a vertical profile as shown in Figure S7(b) (magenta line). Finally, we computed the kink point in the vertical profile where it is possible to define the division of both layers. FL indicates the flowing layer; while SL indicates the solid-like layer.

With the vertical position obtained from the previous procedure, we calculated the depth of the avalanche layer (Fig. S7(b)), as well as mean values of vertical velocities(Fig. S8), packing fraction in both layers(Fig. S9).

V. Final state of force networks for all the tested cases. Figure S11 shows the network of force chains for mixtures of spheres and cubical particles at t=140s of simulation.

VI. Sizes of grains used in the numerical simulations. In the case of cubes made from bonded spheres, the size detailed in Tables S2 and S3 is the diameter of one single sphere. (Note that each cube contain 8 spheres).

Table S1. Size distribution of mixtures of spheres.

Small Grains Diameter (mm)	Big grains Diameter (mm)	Volume Ratio ...
3	3.3	1.3
3	3.6	1.7
3	3.9	2.2
3	4.5	3.4
3	5.1	4.9
3	6	8
3	6.9	12.2
3	8.1	19.7
3	9.3	29.8

Table S2. Size distribution of mixtures of cubes.

Small Grains Diameter (mm)	Big grains Diameter (mm)	Volume Ratio ...
1.5	1.65	1.3
1.5	1.8	1.7
1.5	1.95	2.2
1.5	2.25	3.4
1.5	2.55	4.9
1.5	3	8
1.5	3.45	12.2
1.5	4.05	19.7
1.5	4.65	29.8

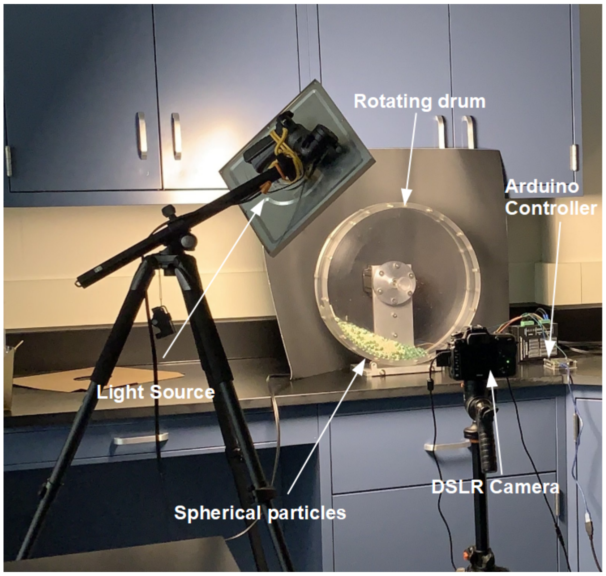

(i)

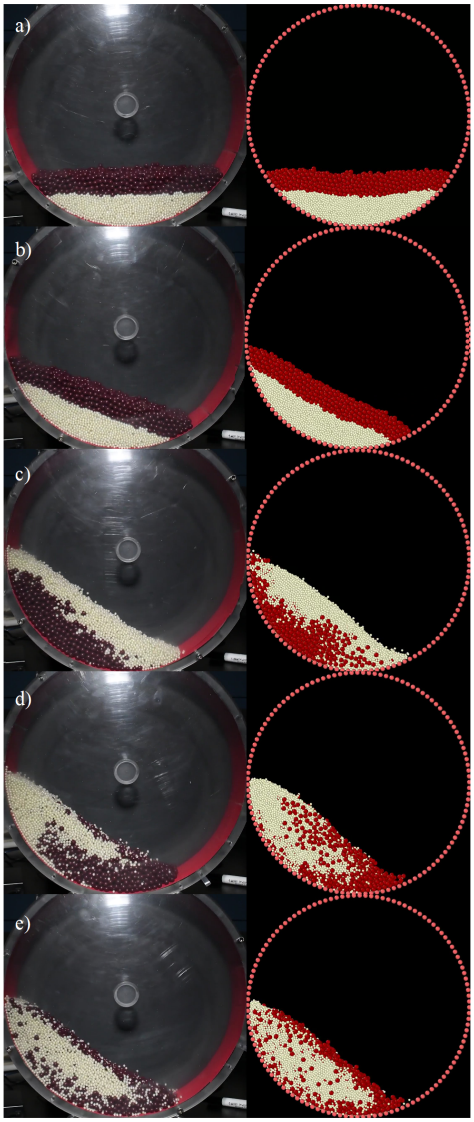

(ii)

Fig. S1. (i) Experimental setup. (ii) Comparison between the experiment (left side of the figure) and the numerical model (right hand of the figure) at: (a) t=0s, (b) t=1s, (c) t=2s, (d) t=4s, and (e) t=8s

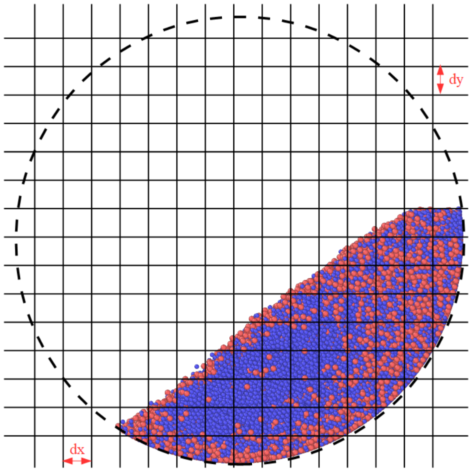

Fig. S2. Scheme of domain and subdomains considered to compute the segregation level

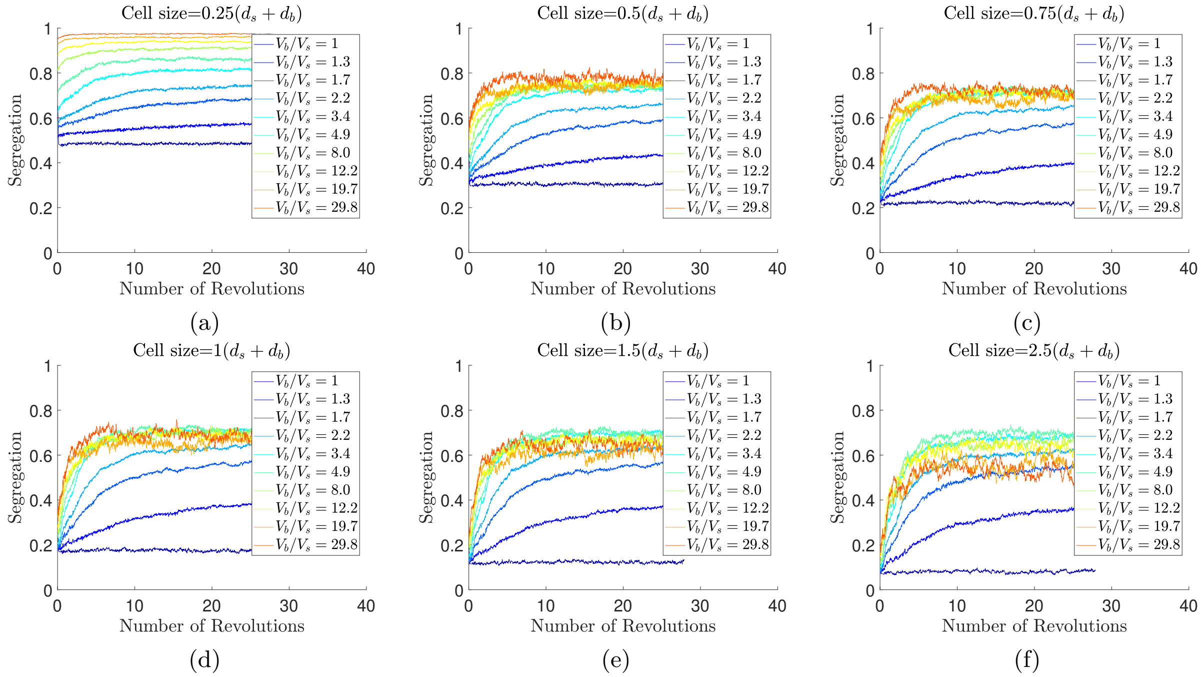

Fig. S3. Temporal evolution of the segregation level for mixtures containing spheres of different sizes with different window sizes. (a) $0.25d_s + d_b$, (b) $0.5d_s + d_b$, (c) $0.75d_s + d_b$, (d) $1d_s + d_b$,(e) $1.5d_s + d_b$, and (f) $2.5d_s + d_b$. Cooler and warmer colors show cases with small and high volume ratios, respectively.

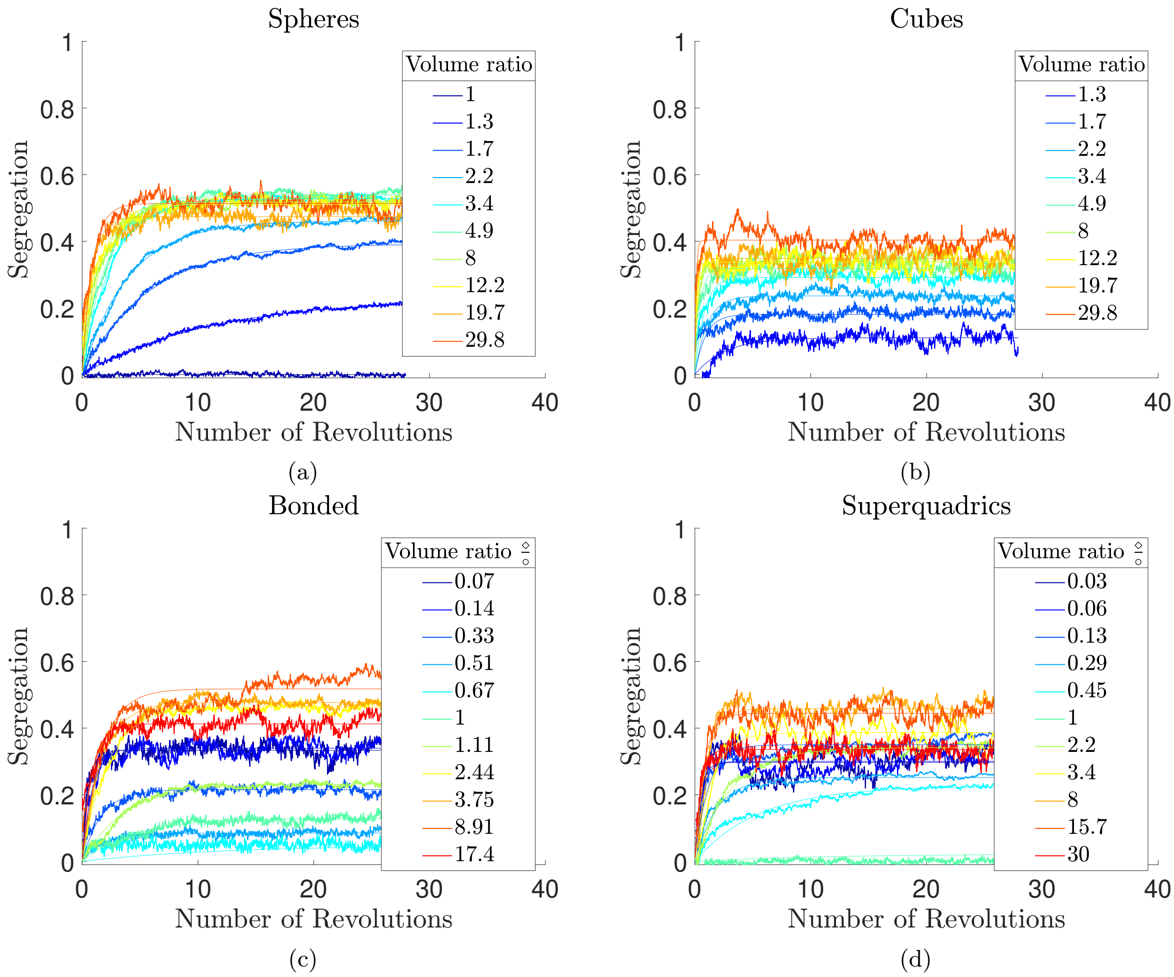

Fig. S4. Temporal evolution of the segregation level for mixtures containing. (a) Spheres of different sizes, (b) cubical bonded particles of different sizes, (c) spheres and cubical bonded particles, and (d) spheres and cubical superquadrics.

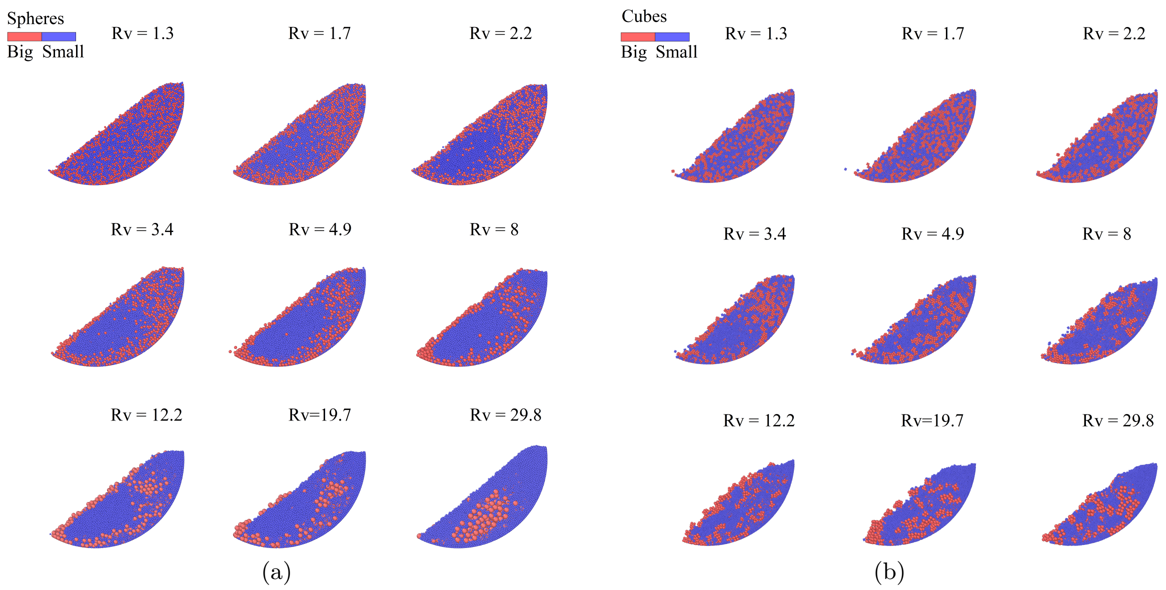

Fig. S5. Snapshots of particle positions for size segregation on: (a) spheres, (b) bonded cubes

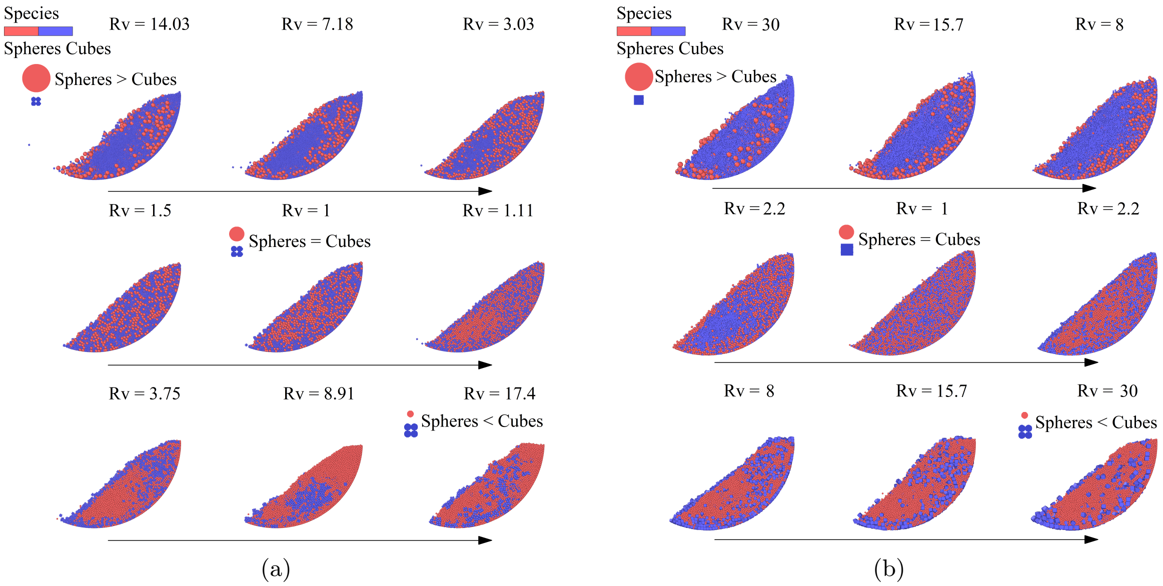

Fig. S6. Snapshots of particle positions for size and shape segregation on spheres and: (a) bonded spheres, and (b) superquadric cubes.

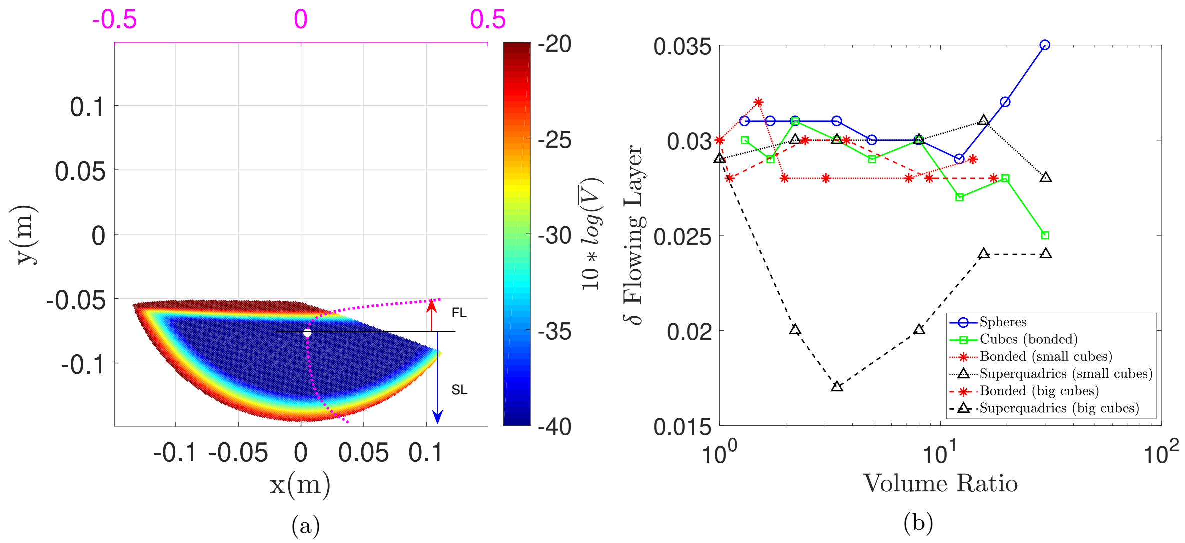

Fig. S7. (a) Scheme of the determination of the depth of the flowing layer in the rotating drum cases. (b) Depth of the flowing layer for all rotating drum cases

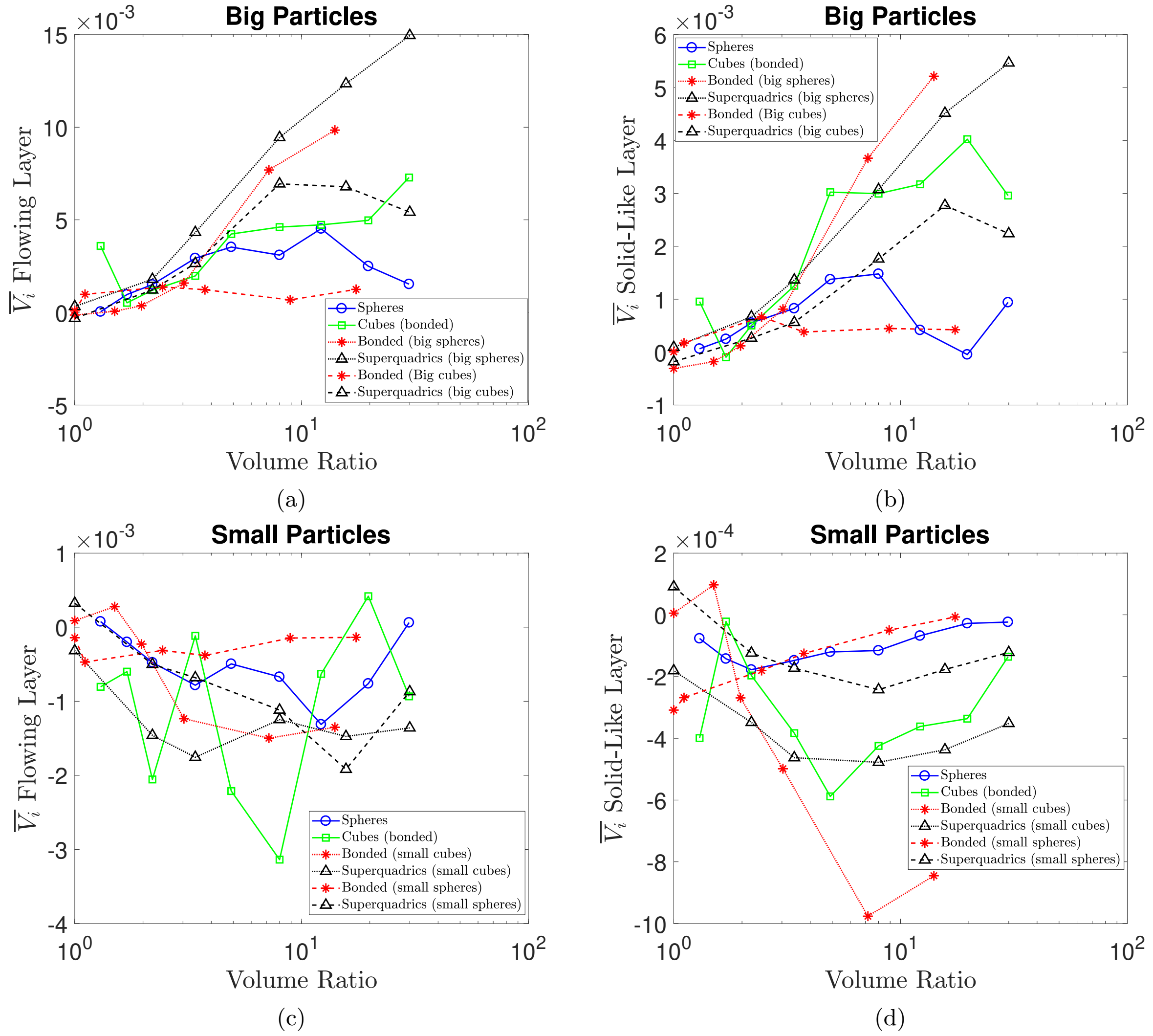

Fig. S8. Mean vertical velocities of particles of: (a) big particles in the flowing layer, (b) big particles in the solid-like layer, (c) small particles in the flowing layer, and (d) small particles in the solid-like layer.

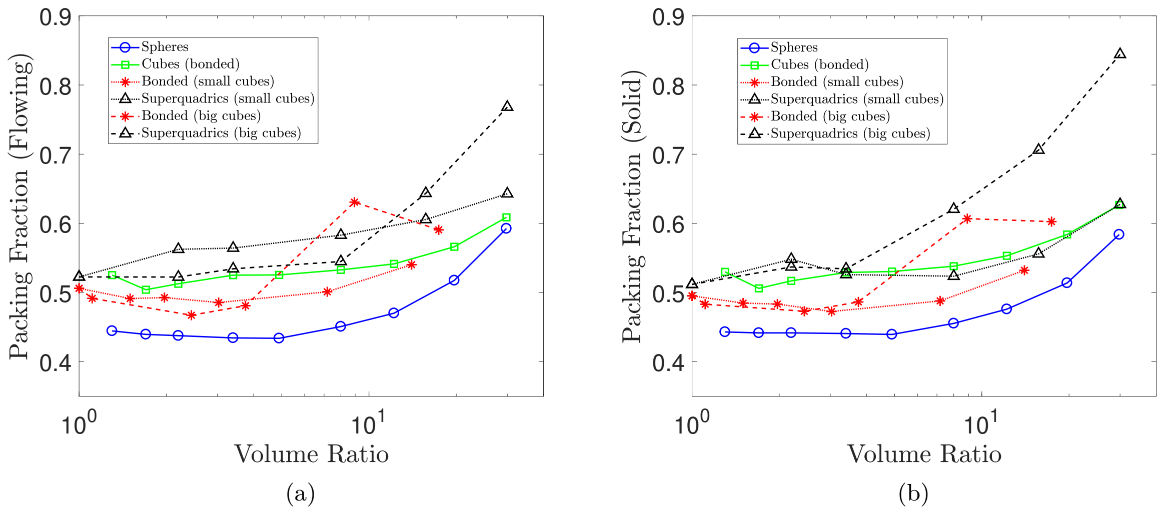

Fig. S9. Mean packing fraction in: (a) flowing layer, and (b) solid-like layer

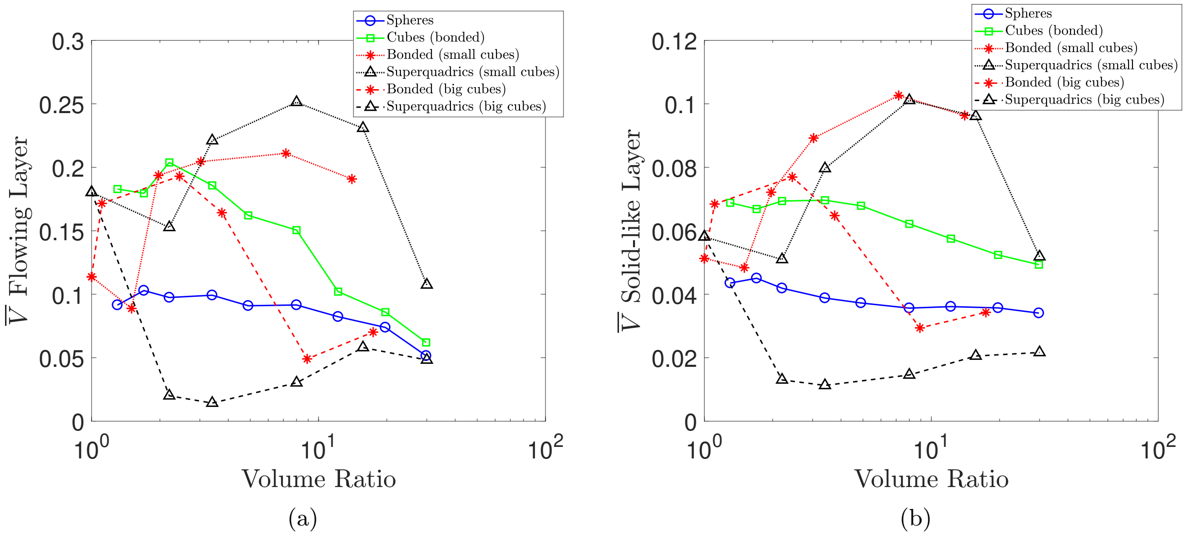

Fig. S10. Mean Velocity of the assembly of grains in: (a) flowing layer, and (b) solid-like layer

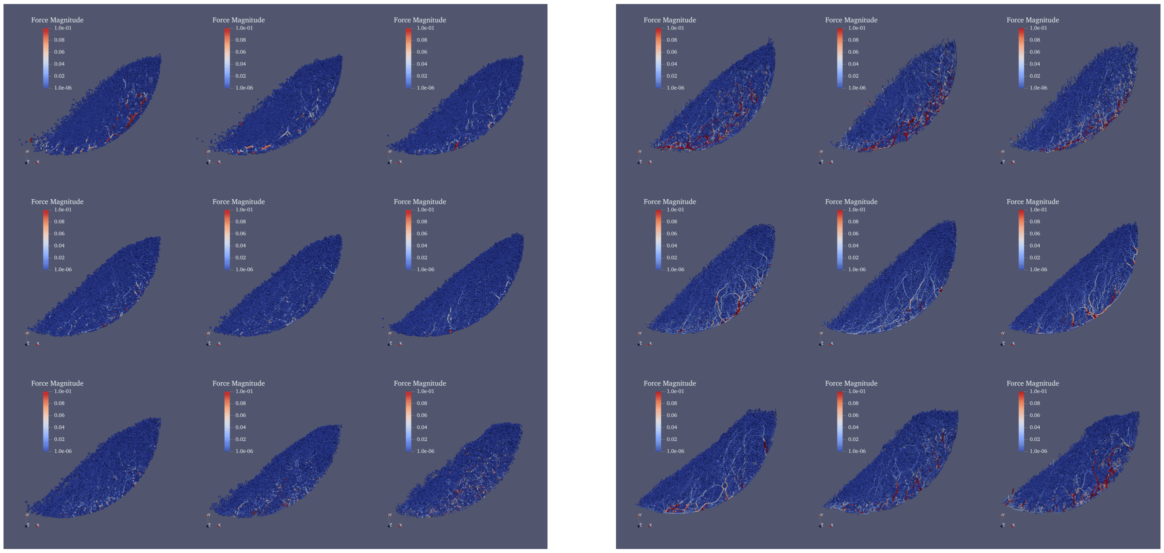

(a) (b)

Fig. S11. Snapshots of network of force chains for size and shape segregation on mixtures with spheres and: (a) bonded particles, and (b) superquadric cubes.

Table S3. Size distribution of mixtures of spheres and cubes (bonded).

Cubes Diameter (mm)	Spheres Diameter (mm)	Volume Ratio $V_\square/V_\circ$
1.5	7.5	0.07
1.5	6	0.14
1.5	4.5	0.33
1.5	3.9	0.51
2	4.7	0.67
2	4.1	1
1.5	3	1.11
1.95	3	2.44
2.25	3	3.75
3	3	8.91
3.75	3	17.4

Movie S1. Validation of the numerical model with an experiment in real time

Movie S2. Numerical model with a mixture of spheres and bonded spheres for the case $V_\square/V_\circ = 0.07$

Movie S3. Numerical model with a mixture of spheres and cubical superquadrics for the case $V_\square/V_\circ = 0.06$

Movie S4. CFD-DEM model of a granular bed composed by spheres and bonded particles and driven by a Couette laminar flow.

Movie S5. Zoom in of a mixture of spheres of different sizes with a volume ratio of 12.

Movie S6. Zoom in of a mixture of cubes of different sizes with a volume ratio of 12.

Movie S7. Zoom in of a mixture of spheres and cubes of different sizes with a volume ratio of $V_\square/V_\circ = 12$**.**

Movie S8. Zoom in of a mixture of spheres and cubes of different sizes with a volume ratio of $V_\square/V_\circ = 0.08$**.**
